# Letter to the Editor: Subjective and objective burden on providers from a multicenter app-based study of patients with cirrhosis and caregivers

**DOI:** 10.1097/HC9.0000000000000137

**Published:** 2023-05-04

**Authors:** Kohilan Gananandan, Rajeshwar Mookerjee

**Affiliations:** 1 Institute for Liver and Digestive Health, University College London, London, UK; 2 Department of Hepatology and Gastroenterology, Aarhus University Hospital, Aarhus N, Denmark

To the editor,

We read with great interest the article by Jawaid et al.^1^ This manuscript tackles an important issue, which is the perceived burden on health care providers of app-based technologies that support clinical decisions. Although the digital transformation is well underway, it is crucial that concerns from care providers, both in terms of their own digital literacy, as well as concerns about potentially increased workload, are addressed to ensure success. We had a few observations, first regarding the Patient Buddy App (PBA) itself and then regarding the survey.

Echoing the feedback from >50% of providers, we have concerns regarding the restrictiveness of the inclusion criteria and the requirement of cohabiting caregivers. Furthermore, patients with alcohol-associated hepatitis were excluded. A retrospective analysis at our own center revealed that this accounts for over 15% of admissions with acute decompensation.^2^ Given this subgroup’s high prevalence, morbidity, and mortality, we feel this exclusion may not accurately reflect the real-world cirrhosis population.

It is difficult in remote monitoring programs to find the balance between maximal data capture and not overburdening the user with time-consuming measurements. PBA assesses a range of parameters but does not seem to monitor physiology (heart rate and its variability and blood pressure), which has been shown to predict impending deterioration and disease progression.^3^ Furthermore, although follow-up in this study was restricted to 30 days, Bajaj et al^4^ and others demonstrate high mortality and readmission rates occurring over 90 days following acute decompensation, suggesting perhaps that the authors should consider extending the PBA program over this at-risk period.

The authors acknowledge that the survey instrument used in this study had not been validated but was developed in accordance with feedback. Regarding the qualitative methods of assessment, although we agree that the questions deployed covered the salient issues, and the Likert scale has been extensively used in research, there are some considerations. Participants’ responses may be influenced by responses to previous questions, with prior demonstration that participants are less likely to pick extremes on such a scale and instead select more central choices.^5^ Instead, one might consider other methods, such as directed interviews and workshops, to garner the opinion of the health care providers. This said, we commend the authors on analyzing the valued feedback from providers to improve the PBA, and we look forward to the results of the randomized controlled trial.
